# Development of an *in vivo* model to study clonal lineage relationships in hematopoietic cells using *Brainbow2.1/Confetti* mice

**DOI:** 10.2144/fsoa-2019-0083

**Published:** 2019-11-18

**Authors:** Jolanda JD de Roo, Sandra A Vloemans, Hans Vrolijk, Edwin FE de Haas, Frank JT Staal

**Affiliations:** 1 Department of Immunohematology & Blood Transfusion, Leiden University Medical Center, Leiden, The Netherlands; 2 Department of Cell & Chemical Biology, Leiden University Medical Center, Leiden, The Netherlands

**Keywords:** *Brainbow* cassettes, cell tracking, *Confetti*, hematopoiesis, lineage tracing, multicolor flow cytometry, retroviral-Cre expression

## Abstract

Hematopoietic stem cells maintain the homeostasis of all blood cell progeny during development and repopulation-demanding events. To study the lineage relationships during hematopoiesis, increasingly complex cell tracing models are being developed. In this study, we describe adaptations to the original *R26R-Confetti* mouse model, which subsequently offers a relatively easy approach to study low complexity clonality during hematopoiesis, with special focus on B and T lymphocyte development. This protocol employs spatiotemporal Cre expression controlled by gammaretroviral transduction for efficient fluorescent protein cell marking. Transplantation of fluorescently marked Lin^-^ cKit^+^ hematopoietic progenitor cells into Rag1^-/-^ mice, resulted in the visualization of differentially contributing stem cell clones to various lineages. Our methodology is useful to study questions in fundamental and preclinical hematopoietic research and *in vivo* B- and T-cell development.

## Lay abstract

Hematopoietic stem cells (HSCs) are responsible for the continuous production of specialized blood cells. B and T cells are blood cells that play an important role in the short and long-term immune system. These cells are frequently replenished, originating from one or multiple HSCs. Our methodology presents a relatively easy way to track the contribution of HSCs or progenitor cells to various mature blood cell types by the use of fluorescent protein cell marking.

Cell lineage tracing is a long-used method to gain knowledge on biological development of cells, tissues, organs and whole organisms [1]. Several methods have been developed to understand stem cell origin and differentiation into other cell types. Many of these methods use molecular markers which require additional purification steps to determine lineage relationships. For instance, viral integration sites and cellular barcoding, all require PCR steps on top of phenotypic analyses. Hence, using fluorescent markers such as green fluorescent protein (GFP) has obvious advantages. By the use of GFP [2] and subsequently altered variations [3–5] new opportunities arose to reliably track cells with fluorescent detection. The *Brainbow* mouse models have introduced an elegant stochastic expression of multiple fluorescent proteins (XFPs) from a single transgene or integration of multiple transgenes to visualize the clonal outgrowth of single cells [6]. The *Brainbow* cassettes have been a useful tool in a wide range of clonal studies for lineage tracing. This toolkit has been frequently adapted and improved with different XFPs, subcellular location tags, XFP protein tags and Cre activity optimization techniques to increase its suitability for a wider range of applications [7–10]. Progressively complex cell tracing systems were developed with the main objective to increase marking resolution to study cell fate mapping [11], but concomitantly the complexity of analysis increases. RGB (Red; Green; Blue) marking is an intricate multifluorescent technique to track cell progeny by the use of simultaneously introduced XFP lentiviral vectors [12], which has additionally combined genetic barcoding to increase detection limits [13]. Since XFPs include limitations in the number or discriminative detection of unique markings, a new artificial DNA recombination locus (Poly-lox) has been recently described allowing the diversity of hundreds of thousands of barcodes for single cell tagging [14].

Cell tracking systems have been interesting tools to study the hierarchical differentiation process of hematopoiesis. All hematopoietic lineages are believed to come from a common ancestor, known as the hematopoietic stem cell (HSC) [15,16]. The current dogma on clonal contribution for prolonged hematopoiesis is an unresolved debate between a reduced number of stable HSCs [17–20] versus a larger number of progenitor cells being the main source for mature blood cells [21–24]. Sun *et al.* developed an *in situ* labeling technique for hematopoietic cells through mobilizing DNA transposons; proposing hematopoiesis is governed by thousands of progenitor cells under physiological conditions [24]. Alternatively, Yu *et al.* studied the clonal contribution of transplanted long-term HSCs (LT-HSCs) with a multifluorescent mouse model (Mx1-Cre; HUe), showing that the majority of the hematopoietic lineages are sustained by merely a few mayor HSC clones [20]. These contradicting results may result from the differing environmental conditions of steady-state versus transplanted HSCs. As an attempt to truly understand HSC contribution, sample-to-sample variance analysis on embryonic and adult hematopoiesis was performed to avoid transplantation induced stress hematopoiesis. By the use of *R26R-Confetti* mice labeled cellular hematopoietic progeny, the frequency of endothelial precursors was calculated for sustained life-long hematopoiesis. Via this approach, Ganuza *et al.* estimated that between 600 and 700 HSC precursors contribute to life-long hematopoiesis [19].

The *R26R-Confetti* mouse was created to study intestinal stem cell fate mapping [25]. The original *brainbow2.1* cassette was combined with a strong CAGG promoter and *LoxP* site in the *Rosa26* locus. This *R26R-Confetti* heterozygous mouse showed a clear stochastic recombination of four fluorescent outcomes (nGFP, YFP, RFP or mCFP) upon *Cre* activation from crossed inducible Cre-mice. Careful analysis of spatiotemporal cell chasing demonstrated to be sufficient to study the intricate differentiation patterns of intestinal stem cells. This model was similarly used as a lower cost and low complexity method to study murine T-cell function and development, although in this study the heterozygous *R26R-Confetti* mouse posed possible marking limitations for T-cell receptor clone analysis [26].

The activation of complex genetic Cre-driven recombination strategies requires an appropriate Cre protein expression. The use of inducible Cre-mice can be difficult to time and persistent Cre activity is potentially toxic [27] or even lethal [28,29]. Additionally, inducible systems for controlling Cre expression [30] can be limiting or insufficient to properly induce all possible fluorescent outcomes in the *Brainbow2.1* model (our own data). Promoter driven *ERT2-Cre* or tamoxifen induced *Cre* recombination in *R26R-Confetti* mice, showed highly underrepresented nGFP and CFP expression resulting in reduced marking [19,26], respectively.

We set out to adapt the *R26R-Confetti* cell tracking model for the study of hematopoietic subsets using murine hematopoietic stem/progenitor cells as target cells. We decided to use viral transduction to introduce the Cre enzyme for XFP recombination. Viral vectors have been developed over the last decades for research and clinical purposes. The improvement of targeting but also transduction protocols have made it relatively easy to target cells of interest under spatiotemporal control [31]. Retroviruses efficiently target HSC and progenitor cells [32,33] and have similar clonal output after transplantation as freshly isolated HSCs [24]. Viral transduction efficiency can be easily adjusted by Cre expression titration and local targeting is ensured for minimal side-effects.

In the present study we show the potential of a homozygous *R26R-Confetti* mouse model in combination with a gammaretroviral *Cre* vector to successfully express 10 XFPs by recombination. We show how to efficiently adopt this model for low complexity fluorescent cell marking combined with lineage tracking using flow cytometry for *in vivo* blood cell lineage tracing studies. This approach allows for efficient recombination of all potential XFPs and sufficient clonal tracking resolution for early B- and T-cell development [20] and other lineage relationships of blood cells.

## Materials & methods

### Mouse cell collection, purification, transduction & transplantation

All mouse procedures were performed with approval from the Leiden University Medical Centre (LUMC) Ethical Committee on Animal Experiments. Male and female homozygous *Gt(ROSA)26Sortm1(CAG-Brainbow2.1)Cle/J* mice (also known as *R26R-Confetti* mice or *R26R-Brainbow2.1* mice; The Jackson laboratory), wild=type *C57Bl/6 Ly5.2* mice and Rag1^-/-^ mice, 6–12 weeks old, were used as donor and recipient, respectively.

*R26R-Confetti* ilium, femurs and sternum were harvested and crushed for bone marrow collection. Fresh or thawed bone marrow were suspended in cold IMDM medium (Gibco, Life Technologies, Bleiswijk, the Netherlands), 2.5% FCS (Greiner Bio-one B.V. Alphen aan den Rijn, the Netherlands) supplemented with 100 U/ml penicillin and 100 μg/ml streptomycin (Gibco, Life Technologies).

Mouse Lin^-^ cKit^+^ (LK) cells were purified by cell sorting on a FACSAriaII cell sorter (BD Biosciences) with the following anti-mouse antibodies: CD3-biotin, Ter119-biotin, GR-1-biotin, B220-biotin, CD11b-biotin, cKit-APC, Sca1-Pe-Cy7 and counterstained with streptavidin-PE (all from eBiosciences, CA, USA). Finally, sorted cells were collected into cold StemSpan serum-free expansion medium (StemSpan-SFEM, StemCell Technologies Inc., Vancouver, BC, Canada) with 100 U/ml penicillin and 100 μg/ml streptomycin Gibco, Life Technologies). Purity was always >95% upon re-analysis.

Sorted LK cells were cultured overnight in StemSpan media (StemCell Technologies) at 37°C 5% CO_2_, with penicillin and streptavidin, 20 ng/ml murine Thrombopoietin (mTPO; a gift from Sanquin, Amsterdam, the Netherlands), 10 ng/ml murine stem cell factor (mSCF; a gift from Amgen, CS, USA), 20 ng/ml mIGF2 (R&D systems, Abington, UK) and 10 ng/ml hFGF1; (a stem cell cytokine cocktail; Prepotech HmbH, Hamburg, Germany) at a concentration of 5 × 10^5^ cells/ml in a 96-well flat bottom plate.

For gammaretroviral transduction, nontissue culture treated 96-well plates (BD Falcon, MA, USA) were coated with retronectin (50 μg/ml, Takara Bio, Inc., Otsu, Japan), subsequently blocked 2% BSA and washed with cold filtered PBS. The desired amount of Integrating Gammaretroviral-Cre-GFP (*iRV-Cre-GFP*) virus was suspended carefully into StemSpan with 100 U/ml penicillin and 100 μg/ml streptavidin and centrifuged within the precoated wells for 1 h at 32°C 900 g. Cultured LK cells were harvested by careful resuspension and counted to determine cell death and cell counts. We aimed at culturing no less than 2.5 × 10^5^ cells/ml per well and carefully added the cells to the centrifuged *iRV-Cre-GFP* loaded wells in complete StemSpan with the above-mentioned stem cell cytokine concentrations.

24 hours after transduction, GPF^+^ cells were sorted and collected in cold StemSpan serum-free expansion medium (StemSpan-SFEM, StemCell Technologies Inc., BC, Canada) with 100 U/ml penicillin and 100 μg/ml streptomycin Gibco, Life Technologies). Purity analysis was always >95%.

The GFP^+^ LK cell fraction was cultured for further analysis at 37°C 5% CO_2_, between 0.5 × 10^5^–2.5 × 10^5^ cells/ml per well in complete StemSpan + the stem cell cytokine cocktail in 96 well round bottom plates (plates (BD Falcon).

### Retroviral vector production, titration & cell line production

Viral particles were produced in low passage 293T cells with 37°C warm complete IMDM medium (Gibco, Life Technologies), 2.5% FCS (Greiner Bio-one B.V. Alphen aan den Rijn, the Netherlands) supplemented with 100 U/ml penicillin and 100 μg/ml streptomycin (Gibco, Life Technologies), transfected with *iRV-Cre-GFP* plasmid [34], *VsVg* envelop plasmid and *pCDNA3 MLV Gag/pol* plasmid. After 24 h culture, 293T cell supernatant was collected and filtered through a 0.22 μm cell filter into prefrozen tubes and stored at -80°C. To extract concentrated *iRV-Cre-GFP* virus, all supernatant was thawed at 4°C and ultra-centrifuged for 16 h at 4°C 10,000 × *g*. Viral supernatant pellets were collected together, supplemented with low volume StemSpan with 100 U/ml penicillin and 100 μg/ml streptomycin. Titers were determined on isolated LK cells to achieve a transduction efficiency of 15-20% in order to minimize Cre-mediated cell toxicity, while maintaining a total of 10 XFP cell marking.

Stable confetti cell lines were developed for fluorescent signal calibration. We transfected single *Brainbow2.1* cassette colors into HeLa cells. HeLa cells were plated into six well plates in about 60% confluence and transfected with either of *Brainbow2.1* plasmids: tandem dimer discosoma red mutant (tdimer2[12]), humanized Renilla reniformis-derived GFP nuclear localization signal (hrGFPnls), monomeric Cerulean (mCerulean) and enhanced yellow fluorescent protein (eYFP). Stable XFP expressing clones were selected using 500 mg/ml G418 sulfate and one FACSAriaII cell sort. The four *Brainbow2.1* XFPs were cloned from CMV-Brainbow-2.1 R (CMV-Brainbow-2.1 R was a gift from Joshua Sanes (Addgene plasmid # 18723; http://n2t.net/addgene:18723; RRID:Addgene_18723) [6] into CMV-Neomycine expressing plasmids.

### Transplantation

Female Rag1^-/-^ mice were kept under specific-pathogen free conditions and treated 1 week before transplantation with triple antibiotic autoclaved acidified drinking water (560 μg/l polymyxin B (Bupha, Uitgeest Netherlands), 700 μg/l ciprofloxacin (Bayer, Mijdrecht, the Netherlands) and 800 μg/l amphotheracin B (Bristol-Myers Squibb, Woerden, the Netherlands). Mice (aged 10 weeks) were lethally irradiated with 8.08 Gy X-rays using orthovoltage irradiation and transplanted 24 h later via tail vein injection with 5000 *iRV-Cre-GFP* transduced (1 day before) GFP^+^ sorted *R26R-Confetti* or wildtype LK cells (Lin^-^ Sca-1^+^) (kept under stem cell conditions) and 10^5^ Rag1^-/-^ spleen cells as myeloid support. Mice were fed Diet gel recovery (Clear H_2_O, Portland, MA, USA) and triple antibiotic water until sacrifice. At 5 or 10 weeks, mice were killed by O_2_/CO_2_ inhalation and peripheral blood (via heart puncture), bone marrow, spleen and thymus were isolated. Bone marrow cell suspension were obtained as previously described, whereas spleen and thymus were homogenized passing through a 70 μm filter. Blood samples were lysed by in-house pharmacy NH4Cl lysis buffer (LUMC, Leiden, the Netherlands).

Part of the transplanted GFP^+^ cells were kept for *in vitro* culturing as described above, to determine XFP recombination efficiency.

### Flowcytometry settings & analyses

All flowcytometry sorts (FACSAriaII cell sort) and measurements (Canto II BD Biosciences or LSRII BS Biosciences) were calibrated first with BD^™^ CompBead Plus, κ/Negative Control (BSA) Compensation Plus (7.5 μm) Particles Set compensation beads (BD Biosciences, CA, USA) and *Brainbow2.1* XFP expressing HeLa cell lines to ensure proper XFP detection and emission spectra distinction. The four *Brainbow2.1* XFPs were detected by the following settings: mCerulean: exc. 407 nm, 450/50 nm bandpass filter; hrGFPnls: 488 nm, em. 505 nm longpass filter, 510/10 nm bandpass filter; eYFP: exc. 488 nm, 525 nm longpass filter, 542/27 nm bandpass filter and tdimer2(12): exc. 561 nm, 600 nm longpass filter, 610/20 nm bandpass filter. For more information on eYFP and hrGFPnls filter sets read the original publication [35].

Flowcytometry analyses were performed using FlowJo software (Treestar, OR, USA). Rarely, manual post-compensation was needed to correct remaining XFP overlap.

### Confocal microscopy analyses

Confocal measurements and analyses were performed on a Leica TCS SP5-AOBS confocal microscope (Leica Microsystems, Wetzlar, Germany) using the following setting: mCerulean: exc. 458 nm, 449-490 nm filter; hrGFPnls exc. 488 nm, 499-511 nm filter; eYFP exc. 514 nm 530-565 nm filter and tdimer2(12) exc. 561 nm 578-615 nm filter. Similarly to flowcytometry, *Brainbow2.1* XFP expressing HeLa cell lines were used for confocal calibration.

Confocal image analysis was performed with Leica LAS AF software. Subsequent automated fluorescent cell detection was performed with an in-house developed program, called Stacks (LUMC). The contrast of individual fluorochrome images was stretched and slightly smoothed to improve uniform intensity within the cells. The four individual fluorochrome images were then merged and a threshold was chosen just above the background peak of the composite gray-value image. To separate closely touching cells, the binary threshold image was eroded with several cycles. Consecutively the eroded image was expanded within the original binary image thereby preventing the separated cells joining each other again. Small objects such as cell debris or dead cells touching the borders were excluded from the measurement this way. The individual cells were then pseudo-color labeled with a unique color upon which the average fluorescent intensity of the four XFPs was measured and exported for further analysis.

## Results

### Confocal ten-color detection

**Figure 1.**
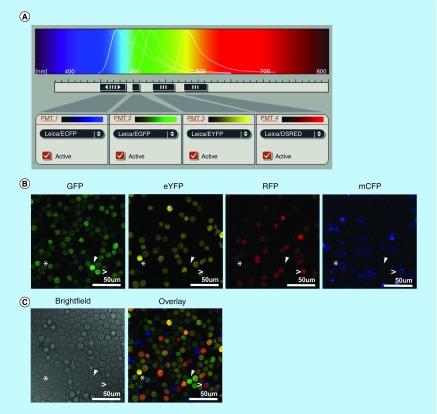
Confocal ten-color detection. **(A)** 4 channels imaged sequentially the *Brainbow2.1* cassette XFPs: mCerulean (exc. 458 nm; 449–490 nm filter), hrGFPnls (exc. 488 nm; 499–511 nm filter), eYFP (exc. 514 nm; 530–565 nm filter) and tdimer2(12) (exc. 561 nm; 578–615 nm filter). **(B)** *R26R-Confetti* LK (Lin^-^cKit^+^) cells were transduced with *iRV-Cre-GFP* and kept under stem cell *in vitro* culture conditions. XFP expression was detected 7 days after transduction solely in *iRV-Cre-GFP* transduced *R26R-Confetti* cells. Untransduced *R26R-Confetti* LK cells never showed XFP expression (data not shown). The images show XFP detection within the same imaging field. Arrows point to GFP-only expressing cells and the asterisks show eYFP-only expressing cells. > signs show double colored RFP/mCFP cells, without GFP viral marking. **(C)** Brightfield and XFP overlay images represent the same imaging section as depicted in A. Single and double-XFP expressing LK cells are shown indicating a high color recombination efficiency of all ten possible XFP outcomes. Additionally, not all colored LK cells show to be GFP positive from the *iRV-Cre-GFP* vector 7 days after transduction. Arrows, asterisks and > signs show the same cellular locations as depicted in the images in A. GFP: Green fluorescent protein; hrGFPnls: Humanized Renilla reniformis-derived GFP nuclear localization signal; eYFP: Enhanced yellow fluorescent protein; mCFP: Membrane tethered mCerulean fluorescent protein; mYFP: Enhanced yellow fluorescent protein; tdimer2(12): RFP: Red fluorescent protein; Tandem dimer discosoma red mutant; XFP: A given fluorescent protein.

To investigate if all theoretical color combinations could be detected in hematopoietic cells, we first transduced *R26R-Confetti* bone marrow LK cells with a gammaretroviral *Cre*-plasmid (*iRV-Cre-GFP*) to initiate XFP recombination. After 7 days *in vitro* culture under stem cell conditions, we observed the expression of four XFPs with confocal microscopy. Using strictly defined spectral band filters, we were able to distinguish different combinations of XFPs (Figure 1A–C). The XFP subcellular compartment labeling was clearly visible for the membrane-bound mCFP (indicated with: >). Due to the *iRV-Cre*-GFP marker expression in the cytoplasm, *Brainbow2.1* hrGFPnls could initially not be distinguished from Cre-GFP. Figure 1C shows exclusively eYFP or GFP marked cells (asterisk or white arrow, respectively), despite the overlapping emission spectra. Less probable XFPs such as RFP and mCFP expression were visible, indicating recombination events on both *Brainbow2.1* cassette alleles (indicated with >).

**Figure 2.**
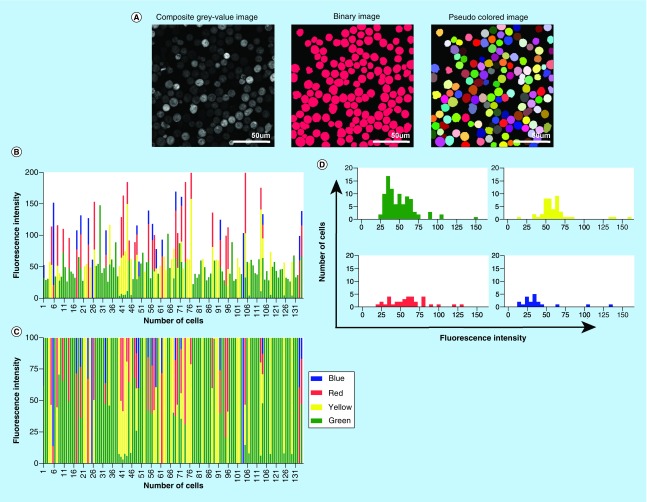
Computational XFP measurement. **(A)** Automated computer detection by an in-house software was performed to objectively measure *R26R-Confetti* XFPs of *in vitro* cultured Lin^-^cKit^+^ cells, 7 days after *iRV-Cre-GFP* transduction. The depicted images are based on the sum of sequential confocal images from each separate XFP; mCerulean, hrGFPnls, eYFP and tdimer2(12). The composite gray-value image is the cumulant of the four XFP individual confocal images with a chosen threshold just above detection background peak for cell definition. The binary image shows the eroded composite gray-value image to ensure proper cell separation. Finally, a pseudo-colored image was generated for visual XFP review, whereby the single XFP images were represented as being respectively green, yellow, red and blue. Software-assigned pseudo-colors originated from the diverse XFP mean fluorescent intensities. **(B)** After computational XFP detection, the mean fluorescent intensity was quantified and visualized per cell as absolute value or **(C)** as percentage of relative XFP contribution of the total (100%). **(D)** Fluorescent intensity patterns were measured by making signal variance graphs for signal threshold detection. A clear threshold was apparent for green, yellow and red. Blue had a less apparent threshold mainly due to less uniform XFP location. Fluorescent intensity patterns are shown per XFP of images in Figure 2A. Interestingly, no higher fluorescent intensity was visible for GFP. At this timepoint, both *iRV-Cre-GFP* and *Brainbow2.1* hrGFPnls can contribute to the total measured green signal. hrGFPnls: Humanized Renilla reniformis-derived GFP nuclear localization signal; mYFP: Enhanced yellow fluorescent protein; tdimer2(12): Tandem dimer discosoma red mutant; XFP: A given fluorescent protein.

Computational detection measured objectively the different XFP expressions. We processed our confocal images through an in-house developed software program for automated cell detection, exclusion of false-positives and XFP detection (Figure 2A). Single-colored cells were used as positive baseline. The average fluorescent intensity of Green; Yellow; Red; Blue was measured in each detected cell resulting in an overall XFP fluorescent intensity expression profile (Figure 2B & C). A range of fluorescent intensities were visible of a maximum of 3 XFP expressions per cell. GFP was always the additional color in the triple-color combination, suggesting its origin from Cre-GFP. Nonetheless, there was no distinct fluorescence intensity distribution from the other XFPs, which is to be expected from the double viral vector (Cre-GFP) and *Brainbow2.1* GFP coloring (Figure 2D).

### GFP expression kinetics

**Figure 3.**
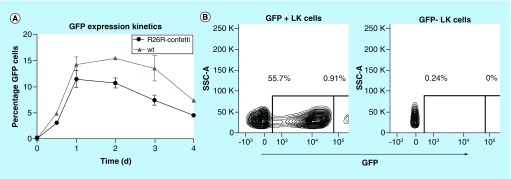
Green fluorescent protein expression kinetics. *R26R-Confetti* Lin^-^cKit^+^ cells were transduced with *iRV-Cre-GFP* and kept under stem cell conditions up to 7 days. **(A)** Green fluorescent protein (GFP) expression kinetics are shown over time. Data are represented as mean ± SD of three mice per group. **(B)** day 7 GFP expression of GFP^+^ and GFP^-^ flow cytometer sorted *iRV-Cre-GFP* transduced *R26R-Confetti* Lin^-^cKit^+^ cells. *Brainbow2.1* cassette hrGFPnls and *iRV-Cre-GFP* could be distinguished by flowcytometry based on their fluorescent intensity. The GFP^hi^ population was not always observed in every *in vitro* experiment. hrGFPnls: Humanized Renilla reniformis-derived GFP nuclear localization signal.

GFP expression was measured by fluorescence-activated cell sorting of *iRV-Cre-GFP* transduced *R26R-Confetti* bone marrow LK cells. Figure 3A shows GFP expression within 24 h while reaching a plateau shortly thereafter. Both *R26R-Confetti* and wildtype (wt) cells lost GFP expression over time which could be due to cytoplasmic transcriptional loss of the nonintegrated *iRV-Cre-GFP* construct. After 7 days culture under stem cell conditions, ±1% highly GFP expressing cells (GFP^hi^) showed to be integration-competent particles as reported previously [34] (Figure 3C). This GFP^hi^ expressed none of the other *Brainbow2.1* XFPs (data not shown). Even though this population was not detected in every sample, this made it possible to distinguish stable integrated *iRV-Cre-GFP* cells from *Brainbow2.1* hrGFPnls expressing cells with flow cytometry analysis. All other XFPs were detectable from 48 h onward (Supplementary Figure 1A) with expected maturation kinetics [36].

### Ten-color detection by flow cytometry

**Figure 4.**
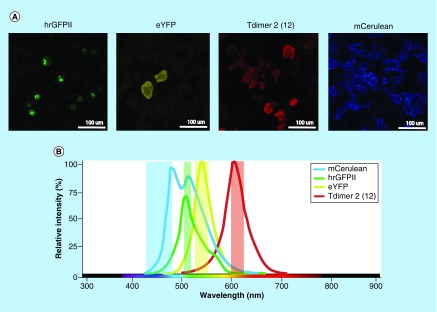

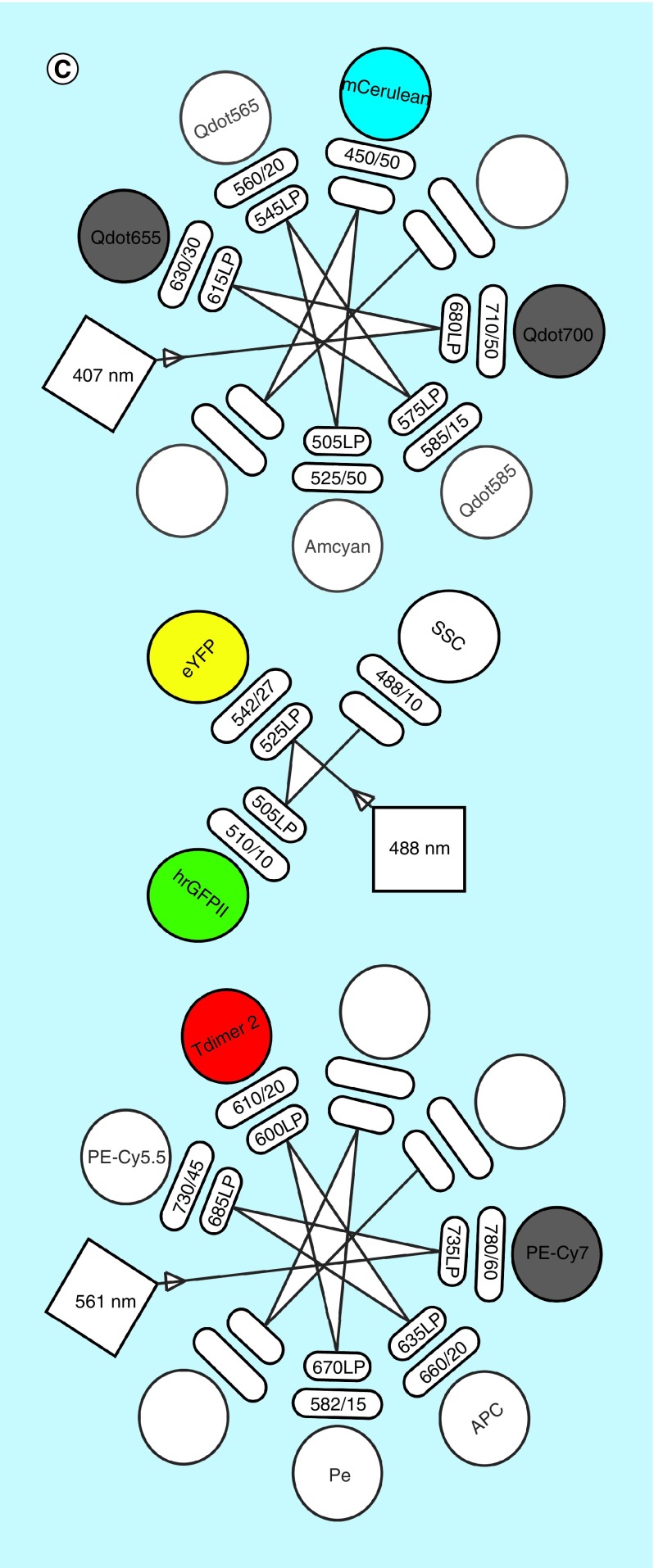

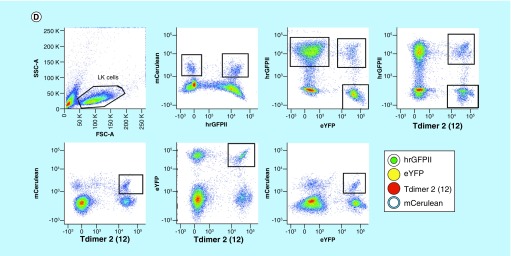
Flowcytometry ten-color detection. **(A)** HeLa cells were stably transduced with *Brainbow2.1* cassette single XFP vectors to calibrate the flow cytometer. Images depict early stages of single-XFP HeLa cell line production. Further XFP expression of these cells was detected by flow cytometry and XFP positive cells were cell sorted to aid higher confluent cell lines. Nuclear localization of hrGFPnls, cytoplasmic localization of eYFP and tdimer2(12) and membrane tethered mCerulean are visible with confocal microscopy. The same excitation values and filter sets were used as mentioned in Figure 1A. **(B)** Visual of the normalized emission spectra of the four *Brainbow2.1* cassette XFPs with the detection ranges of our proposed flowcytometry filter set. **(C)** Flow cytometer filter strategy for proper 10-color detection without XFP signal spillover. mCerulean: exc. 407 nm, 450/50 nm bandpass filter; hrGFPnls: exc. 488 nm, 505 nm longpass filter, 510/10 nm bandpass filter, eYFP: exc. 488 nm, 525 nm longpass filter, 542/27 nm bandpass filter and tdimer2(12): exc. 561 nm, 600 nm longpass filter, 610/20 nm bandpass filter. **(D)** Flowcytometry analysis gating strategy of *R26R-Confetti iRV-Cre-GFP* transduced Lin^-^cKit^+^ cells. The fluorescence-activated cell sorting plots depict an exemplary *Brainbow2.1* XFP expression analysis of Lin-cKit+ cells after 7 days stem cell conditioned *in vitro* culture. Clean separation and measurement of the 10 *R26R-Confetti* XFPs is shown. Exclusion of other XFPs was performed in the gating strategy for every single or double XFP to ensure pure populations. No bleed-through phenomena have been found between GFP/eYFP nor GFP/mCFP (data not shown). hrGFPnls: Humanized Renilla reniformis-derived GFP nuclear localization signal; eYFP: Enhanced yellow fluorescent protein; tdimer2(12): Tandem dimer discosoma red mutant.

We established stable XFP expressing cell lines to properly distinguish the ten expected XFP color combinations in the cells of interest. HeLa cells transfected with one of four *Brainbow2.1* color-encoding vectors were used to calibrate our flow cytometer. Nuclear localization of hrGFPnls, cytoplasmic localization of eYFP and tdimer2(12), and membrane tethered mCerulean were visible in the acquired confocal images of single XFP expressing HeLa cells (Figure 4A). Figure 4B & C illustrates the XFP emission spectra and chosen longpass and bandpass filters for clean XFP emission detection within a flow cytometric system. Our proposed flow cytometer filter strategy offers possibilities to expand the filter set for fluorescent detection of surface characterization markers without fluorescence contamination. Accurate separation of ten XFP combinations in *iRV-Cre-GFP* transduced *R26R-Confetti* bone marrow LK cells was achieved (Figure 4D). Even though GFP and eYFP were both exited by 488 nm, no fluorescence bleed-through was visible. The chosen filter combination for GFP and eYFP provides the advantage of clean distinction between these largely overlapping fluorophores [35]. Our filter strategy facilitates combining Confetti color cell tracking with cell surface antibody characterization and possibilities to sort individual cells for further culturing or molecular analyses.

### *In vivo* XFP expression

**Figure 5.**
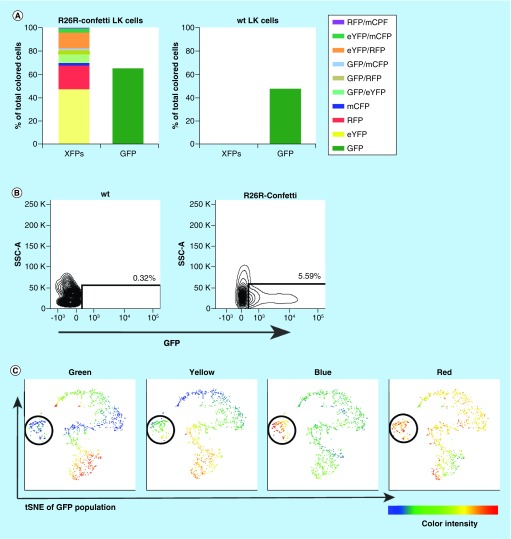

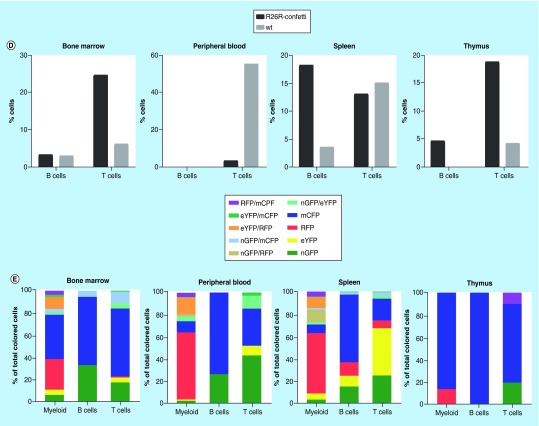
*R26R-Confetti in vivo* hematopoietic reconstitution. Rag1^-/-^ mice were transplanted with *R26R-Confetti* or wt Lin^-^cKit^+^ (LK) GFP^+^ (*iRV-Cre-GFP* transduced) sorted LK cells. *Ex vivo* data in this figure are a representative example out of three analyzed mice. Bone marrow, spleen, peripheral blood and thymus were analyzed for T cell, B cell development and *R26R-Confetti* XFPs by flow cytometry. **(A)** 7-day *in vitro* culture of transplant material showing XFP distribution and GFP expression (either from *iRV-Cre-GFP* or a mixture with *Brainbow2.1* cassette hrGFPnls) of transplanted material. All 10 XFP combinations were expressed within our LK *iRV-Cre-GFP* transduced population. **(B)** Example of flow cytometer peripheral blood GFP measurement of *R26R-Confetti* (5 weeks posttransplant) or wt (10 weeks posttransplant) Rag1^-/-^ mice (gated from total life cells). No GFP was detected in any wt transplant Rag1^-/-^ mouse nor in any of the studied organs (bone marrow, spleen, peripheral blood and thymus). **(C)** tSNE cluster analysis of GFP^+^ peripheral blood cells of *R26R-Confetti* transplant Rag1^-/-^ mouse, showing not all cells are GFP^+^ while the same cluster is positive for the other *R26R-Confetti* XFPs (mentioned in picture header). For the tSNE cluster analysis a dimensional reduction of 5000 events was chosen. The encircled cluster was defined as predominantly GR-1 expressing myeloid cells. **(D)** E*x vivo* organ reconstitution data of B and T cells of *R26R-Confetti* transplant (5 weeks sacrifice) and wt transplant (10 weeks sacrifice) Rag1^-/-^ mice. **(E)** Concordant *R26R-Confetti* transplant Rag1^-/-^ mouse data from Figure 5D showing 10 XFP expression patterns of *ex vivo* organs (5 weeks sacrifice). *Brainbow2.1* hrGFPnls is here referred to as nGFP. Exclusion of other XFPs was performed in the gating strategy for every single or combination XFP to ensure pure populations. eYFP: Enhanced yellow fluorescentprotein; GFP: Green fluorescent protein; hrGFPnls: Humanized Renilla reniformis-derived GFP nuclear localization signal; mCerulean: Monomeric cerulean; mCFP: Membrane tethered mCerulean; RFP: Red fluorescent protein referring to tdimer2(12); tdimer2(12): Tandem dimer discosoma red mutant; XFP: a given fluorescent protein.

We used the *R26R-Confetti* mice and wt mice for clonality detection after transplantation of sorted GFP^+^ (*iRV-Cre-GFP* transduced) LK cells into lethally irradiated T-and B-cell deficient RAG1^-/-^ mice (n = 5; 3 *R26R-Confetti mice* and two wt mice LK cell transplantations). We chose LK cells as progenitor population for our experiments for myeloid supported short-term engraftment visualization. LK cells have equal potential to reconstitute B and T cells shortly after transplantation as Lin^-^ Sca1^+^ Ckit^+^ (LSK) HSCs [37]. Reconstitution and clonal XFP kinetics were unique in every mouse, therefore we chose to display one exemplary mouse dataset in Figure 5. All XFP combinations were expressed in the *in vitro* cultured GFP^+^ sorted LK cells, which gave a reflection of what to expect *in vivo* (Figure 5A). GFP at 7 days *in vitro* culture could not be distinguished from viral Cre-GFP or *Brainbow2.1* hrGFPnls (further referred to as nGFP), therefore we refer it as GFP and depict it separate from all other XFPs in this figure. *R26R-Confetti* and wt stem cells gave rise to B cell and T cells in the RAG1^-/-^ mice after transplantation (Figure 5D). *R26R-Confetti* transplanted mice were sacrificed at 5 weeks (wt at 10 weeks), showing T and B cells repopulation per organ at early reconstitution. Interestingly, no GFP^hi^ cells were detected in the *R26R-Confetti* and wt transplanted RAG1^-/-^ mice indicating a lack of outgrowth of the small fraction of LK cells with integrated *iRV-Cre-GFP* (Figure 5B). This suggests that all GFP expressing cells originate from the *R26R-Confetti* transgene nGFP. In the case Cre-GFP would still be present in the progeny cells, all colored cells should express GFP. We examined this plausibility of simultaneous GFP, eYFP, RFP and mCFP expression by T-distributed Stochastic Neighbor Embedding (t-SNE) analysis. Not all eYFP, RFP and mCFP expressing clusters were positive for GFP expression (Figure 5C), which would be expected for integrated *iRV-Cre-GFP* cell progeny. The miniscule cluster of triple-positive marking consisted out of cell debris.

Interestingly, a reflection of early B- and T-cell reconstitution can be seen in the XFP expression of the *R26R-Confetti* LK transplanted RAG1^-/-^ mice (Figure 5E). B cell development starts at the bone marrow, after which CD19 expressing immature B cells migrate to the spleen for complete maturation. *R26R-Confetti* labeling efficiencies of total B cells differed per organ (±1,6% in bone marrow; ±1,3% in spleen and ±74,4% in peripheral blood). Spleen B cells contained six different XFP's (±15% nGFP, ±10% eYFP, ±10% RFP, ±55% mCFP, ±5% nGFP/mCFP and ±1%e YFP/mCFP), whereas 3 XFP's (±35% nGFP, ±55% mCFP and ±5% nGFP/mCFP) could be detected in bone marrow B cells. Only nGFP (±30%) and mCFP (±70%) expressing B cell clones were detected in peripheral blood, suggesting that most of the early reconstituting B cell clones which have homed to the spleen are no longer present in the bone marrow.

T-cell reconstitution showed a similar diminishing XFP expression patterns seemingly dependent on location and developmental timing. Here, *R26R-Confetti* labeling efficiency of total CD3^+^ T cells in the thymus was ±7,2%, whereas for thymic CD3^-^ pre-T cells it was ±23,30%. Peripheral blood had ±11,90% labeled cells of total circulating T cells. Thymocytes originated from three different XFP expressing clones (±20% nGFP, ±70% mCFP and ±10% RFP/mCFP) that ultimately exit the thymus, whereas more XFP clones were visible in the peripheral organs. Bone marrow and spleen T cells were composed out of the same XFP expressing clones (nGFP, eYFP, RFP, mCFP, nGFP/eYFP, GFP/mCFP and eYFP/mCFP), but the relative distribution of these clones was different between the organs. eYFP and RFP T cells were for example much more represented in the spleen than in the bone marrow (eYFP ±40% vs. ±5%; RFP ±10% vs. ±2%, respectively), whereas mCFP T cells were more present in the bone marrow than in the spleen (±60% vs. ±10%, respectively). Interestingly, the thymus contained an RFP/mCFP expressing subset which was not found in the other organs.

## Discussion

The use of fluorescent cell lineage tracing models has recently gained momentum due to the continuous improvements in new fluorescent proteins and emerging applications [38]. Smarter XFP strategies are recommended to diminish spectral overlap or have enhanced fluorescent intensity for easier detection [39]. Additionally, guidelines on how to choose the right XFP to ensure appropriate fluorescence detection [40] have become indispensable for XFP model development. The purpose of this study was to optimize the *R26R-Confetti* mouse model to its full potential by the use of a Cre-retrovirus and carefully defined fluorescent detection strategies. *In vivo* XFP expression allowed the study of early reconstitution in the hematopoietic system by short-term lineage tracing. Our approach shows the possibility of enhancing an existing cell tracing model with a more efficient Cre recombination method to study hematopoietic lineage questions resulting in a relatively easy and low-cost manner.

Previous *R26R-Confetti* or similar multifluorescent model studies (Mx1-Cre;Hue) were unable to benefit from all XFP combinations most likely due to inadequate Cre enzymatic activity [19,20,26]. Conditionally expressed Cre models (*ERT2-Cre*) or promoter-driven Cre expression have shown to be suboptimal with relatively low XFP marking. Furthermore, repetitive and long administration of chemical agents or timely uncontrolled Cre activation can additionally lead to overactive XFP recombination [6]. Retroviral transduction yields a wide scale of transduction efficiencies with the possibility to adjust the multiplicity of infection and thereby expression levels. The used retroviral *iRV-Cre-GFP* vector showed a rapid onset of GFP expression and efficient recombination of all *Brainbow2.1* XFPs within 7 days after transduction at a relatively efficient infection rate. Even though this retrovirus is integration proficient, no long-term integration effects were detected after 5- (*R26R-Confetti* material) or 10-week (wt material) transplantation. We detected a population of ±1% high GFP expressing cells probably from proviral DNA, 7 days after *iRV-Cre-GFP* transduction, yet these cells did not survive or repopulate the RAG1^-/-^ mice after transplantation. This could be due to stress hematopoiesis [41], strong selective pressure during progenitor homing or decreased cell viability of high Cre-expressing cells [27]. In several multifluorescent cell tracking methods, subcellular compartment labeling has proven to be useful for clonal analysis by imaging techniques [7,10,42]. Interestingly, the lack of *iRV-Cre-GFP* integration made it possible to detect *R26R-Confetti* nGFP via flow cytometry, without having to rely on confocal microscopy for nuclear localization. Alternatively, a different fluorescent viral marker could avoid misinterpretations of the GFP origin in the *R26R-Confetti* model. A Far-red XFP with a high maturation rate and protein/mRNA degradation signal [43] for rapid XFP detection and breakdown could be an interesting candidate for studies in highly autofluorescent bone marrow [44,45].

In our study, XFP expression was not distributed near-equal ratio as reported by *Snippert et al.*, [25] nor was nGFP expression underrepresented after longer *in vivo* cell tracking. In fact, our data reports a low fraction of double XFP expressing cells following Cre expression, indicating a decreased XFP expression probability according to recombination event complexity. Additionally, we show preference for mCFP expressing clones after prolonged *in vivo* cell tracking (5 weeks chase period). Even though the *R26R-Confetti* starting material expressed all 10 XFP combinations, either selective repopulation or overactive Cre expression could cause this mCFP monochromacy. Considering that LK cells are enriched for myeloid progenitors and only a small proportion have true long-term stem cell capacity, the observed monochromatic XFP expression could in part be imputable to clonal extinction of short-term progenitors. Snippert *et al.*, confirmed intestinal crypt clonicity after detecting monochromatic (eYFP:RFP:mCFP) or even fully unlabeled crypts derived from LGR5^hi^ stem cells between 2 weeks to 2 months’ timeframe, but no overrepresentation of mCFP expression was mentioned [25]. To date no cases have reported positive selective recombination of mCFP expression. On the contrary, mCFP tends to show low protein expression, requiring a secondary reagent for detection [26]. Our data do not have any of the aforementioned XFP expression limitations, demonstrating efficient gammaretroviral Cre expression for complex recombination strategies.

Continuous Cre expression can reduce the *Brainbow2.1* construct to single or XFP-null DNA recombination [6], suggesting a needful balance between Cre recombination efficiency and duration. Not only unwanted XFP excision but also cytotoxicity is determining for an optimal working confetti model. Several reports have demonstrated disadvantageous effects of prolonged or overactive Cre expression, such as growth inhibition or genotoxicity [27]. De novo-synthesized *iRV-Cre-GFP* RNA could be reduced by shRNAs but was shown to be merely partially effective [34]. To better control the Cre activity time window, an integrase-deficient gammaretroviral Cre vector SF91aPBS.nlsCre [34] could be an interesting alternative compared with our vector system even though this vector has a slightly lower Cre recombination efficiency at low concentration. Alternatively, a self-excising Cre [46] modification could be employed to the *iRV-Cre-GFP* construct to control recombination duration. This would be an interesting improvement for future studies without the concern of high transduction toxicity levels.

For *in vivo* stem cell potency studies, LSK cells have been efficiently transduced with either retro- or lentiviruses while still being able to efficiently repopulate immunodeficient mice. Transplantation causes stress hematopoiesis which initiates a strong proliferative pressure on transplanted cells [19]. Only a few major clones (making up 80% of all hematopoietic cells) are believed to sustain hematopoietic reconstitution while most clonal changes proceed up to 2–3 months post-transplantation. In that study only 5–11 clones were detected to contribute to hematopoiesis, dismissing the need of their 10^3^ multifluorescent marking capacity model [20]. We show the applicability of *iRV-Cre-GFP* transduced *R26R-Confetti* LK progenitor cells for the study of early hematopoietic reconstitution. The developmental pathways of B and T cells were reflected in the bone marrow, spleen and thymus, respectively. Early developing B cells start in the bone marrow and migrate to the spleen for further maturation [47]. Our data showed a diminishing clonal XFP pattern according to this migration process. Nonetheless, the thymus contained an XFP clone which was not detected in egressed mature T cells, suggesting a continuously colonized thymus by new bone marrow-derived progenitors [48] and confirming our previous notion using lentiviral barcoding that only a small fraction of HSC clones contributes to thymic development and the T-cell lineage [49]. Pre T cells (lacking CD3 expression) in the thymus showed a different XFP pattern including an RFP clone (Supplementary Figure 1B). This XFP clone was not detected in thymic CD3^+^ mature T cells although present in the periphery, illustrating the restrictive capacity of the thymus but also new thymic seeding of possibly the same hematopoietic progenitor. Previous data showed similar clonality patterns of T-cell development by positive and negative selection, which are known to be important check-points of clonal restriction [49].

While our initial question was how to optimize the homozygous *R26R-Confetti* mouse model for XFP FACS detection methods, our data confirm the applicability of this model for early hematopoietic reconstitution studies. The current form of our method may not be suitable for long-term contributing stem cells, but gives information on a limited number of hematopoietic clones over a short time period. LK cells have shown to easily adapt confetti cell marking, partially due to their higher cell cycling rate among HSC populations [50]. LSK cells were similarly able to efficiently recombine all *Brainbow2.1* colors *in vitro* (data not shown), but have not been studied in our *in vivo* model. Yu *et al.*, alike us combined a multifluorescent model with extracellular markers for flowcytometry analysis, yet were unable to use the full XFP marking potential [20]. Multicolor flow panel techniques are being developed continuously and will give more options to combine a broad scale of XFP expression with phenotypic cell characterization. The *R26R-Confetti* model with retroviral Cre expression offers the possibility to perform low complexity and low-cost B- and T-cell clonality experiments. Noteworthy is that also tissue architecture studies could be performed with confocal imaging for the study of intrathymic migration [51–53] of developing T cells [54] or B-cell development and maturation within bone marrow and spleen [55], which are to date poorly understood.

## Future perspective

HSC differentiation patters have been studied extensively to understand their contribution to the hematopoietic homeostasis. Much effort has been employed for stem cell identification, and now cell tracking techniques offer the possibility to follow their progeny. While the study of low dividing stem cells and their diverse progeny is thought to require complex genetic cell marking to increase marking resolution, researchers should also consider the increasing complexity of resulting datasets. The challenge for upcoming years is to combine cell tracking studies with the extensive and still expanding knowledge of stem cell defining factors. Quantum computing can play an important role in the analysis and final understanding of these multidimensional research questions.

**Summary points**

- Low complexity B- and T-cell clonality in hematopoietic reconstitution can be studied by the homozygous *R26R-Confetti* mouse model.
- Higher number fluorescent protein lineage tracing is possible by flow cytometry with an appropriate emission spectra separation filter strategy.
- Confocal microscopy and flow cytometry are reinforcing techniques to understand lineage tracing models.
- Gammaretroviral transduction is an elegant tool to control spatio-temporal Cre expression in hematopoietic stem/progenitor cells, while avoiding toxicity.
