# Supplementary figures and images for: Development of an in vivo model to study clonal lineage relationships in hematopoietic cells using Brainbow2.1/Confetti mice
Source: Future Sci OA. 2019 Nov 18;5(10):FSO427. doi: 10.2144/fsoa-2019-0083 (PMC6900974; doi:10.2144/fsoa-2019-0083)

# Supplemental Figure

A

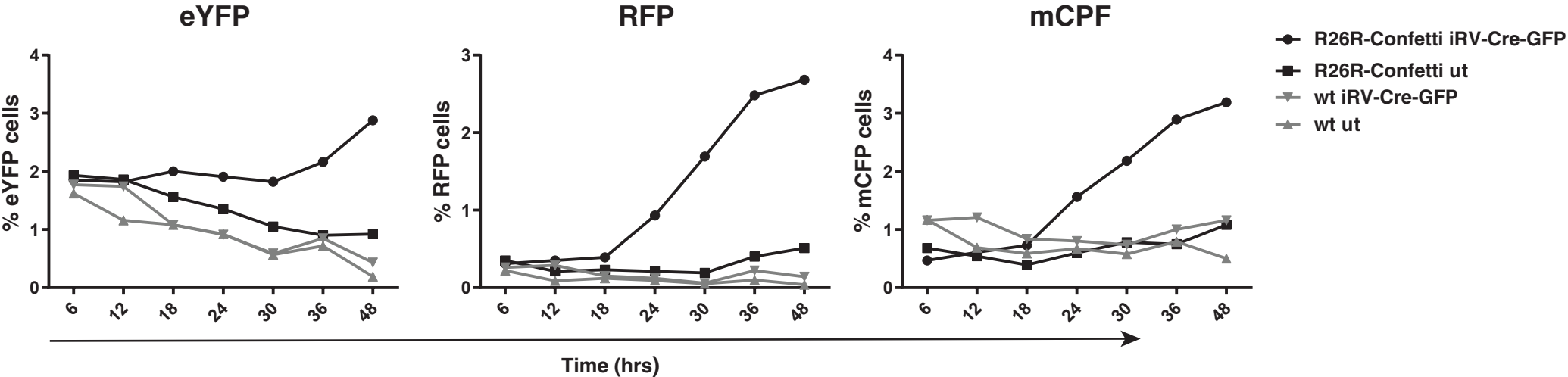

B

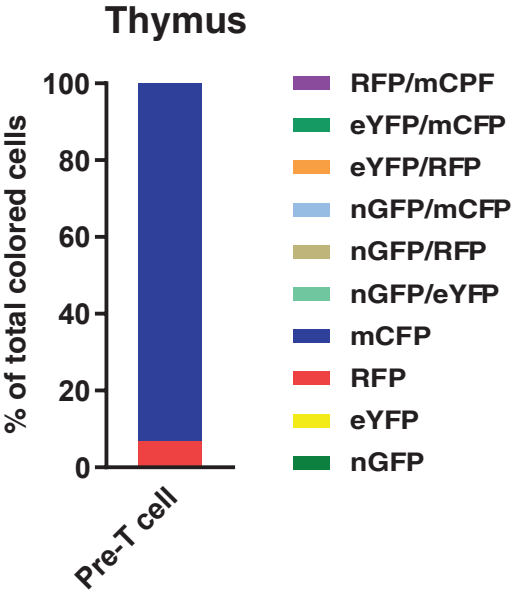

Supplement: Supplementary file 1 [file fsoa-05-427-s1.pdf]
